# New Disk Carrier

**Published:** 1883-10

**Authors:** 


### NEW DISK CARRIER.

Some time since we purchased from Hood & Reynolds a new form of carrier for the sand-paper disks now so extensively used. The old parting nut or screw-head mandrels demanded too much time to make the frequent changes necessary. The disk carrier under consideration has a flat face, from which project four claw-shaped points, upon which the disk is forced by a small self-centering cylinder, and these effectually retain it, while it may be removed with a single motion. We have found them to be a great convenience.

